# Prevalence and characterization of *Clostridium perfringens* toxinotypes among patients with antibiotic-associated diarrhea in Iran

**DOI:** 10.1038/s41598-019-44281-5

**Published:** 2019-05-24

**Authors:** Masoumeh Azimirad, Fatemeh Gholami, Abbas Yadegar, Daniel R. Knight, Sharareh Shamloei, Hamid Asadzadeh Aghdaei, Mohammad Reza Zali

**Affiliations:** 1 Foodborne and Waterborne Diseases Research Center, Research Institute for Gastroenterology and Liver Diseases, Shahid Beheshti University of Medical Sciences, Tehran, Iran; 2 School of Veterinary and Life Sciences, Murdoch University, Murdoch, Western Australia Australia; 3 Department of Water and Wastewater Quality Control Laboratory, Water and Wastewater Company, Tehran, Iran; 4 Basic and Molecular Epidemiology of Gastrointestinal Disorders Research Center, Research Institute for Gastroenterology and Liver Diseases, Shahid Beheshti University of Medical Sciences, Tehran, Iran; 5 Gastroenterology and Liver Diseases Research Center, Research Institute for Gastroenterology and Liver Diseases, Shahid Beheshti University of Medical Sciences, Tehran, Iran

**Keywords:** Bacteriology, Gastrointestinal diseases

## Abstract

*Clostridium perfringens* has emerged as an important cause of antibiotic-associated diarrhea (AAD), particularly in the hospital environment. Here we investigated the prevalence and molecular epidemiology of *C. perfringens* isolated from 2280 fecal samples from Iranian diarrheal patients suspected of having AAD. Overall, AAD was diagnosed in 13.3% (303/2280) of patients and associated with advanced age (>50 years, *P* = 0.001). A total of 106 *C. perfringens* isolates were cultured from AAD (*n* = 68) and non-AAD (*n* = 38) groups, with toxinotypes A and F comprising 84% and 16% of isolates, respectively. Notably, 41.2% of type F strains were also *cpb2*-positive and enterotoxigenic *cpe*-positive strains were detected in 13.2% of the isolates from AAD patients. Genes associated with the VirR/VirS signal transduction (*virR*, *virS*) and accessory gene regulator (*agrB*, *agrD*) systems were detected in 56.6% and 67% of the isolates, respectively, and peptides of the quorum-sensing modulator, AgrD were highly conserved across all strains. The high prevalence of *C. perfringens* in Iranian AAD patients suggests that diagnostic laboratories in this region should consider screening for *C. perfringens* in cases of suspected AAD, especially if the specimen is negative for other pathogens and/or the patients are aged >50 years.

## Introduction

Antibiotic-associated diarrhea (AAD) is a serious complication that remains a major healthcare problem, in both hospitalized patients and in the community^1^. *Clostridioides difficile* is a well-known etiologic cause of AAD, accounting for 10–33% of all cases^2,3^. However, other bacterial pathogens including *Clostridium perfringens*, *Staphylococcus aureus* and *Klebsiella oxytoca* have been implicated in the development of AAD^2–4^. *C. perfringens* is a ubiquitous Gram-positive spore-forming anaerobe, which can cause numerous diseases in humans including food poisoning, gas gangrene, enteritis necroticans, sudden death syndrome, and enterotoxemia^5,6^. In 1984 Borriello *et al*. was the first to report *C. perfringens* as a cause of AAD in patients with nosocomial diarrhea^7^, and many studies have suggested a crucial role for *C. perfringens* in diarrheic patients with a history of antibiotic usage^8,9^. It has been estimated that up to 15% of all AAD patients were infected with enterotoxigenic *C. perfringens*^3,10^. *C. perfringens* has historically been classified into five toxigenic types (A-E) depending upon its ability to produce four major exotoxins including α-, β-, ɛ-, and ɩ-toxin encoded by *cpa*, *cpb*, *etx*, and *iap/ibp* genes, respectively^11,12^. However, based on a recently introduced toxin-based typing system, *C. perfringens* is now reclassified into seven toxinotypes (A-G)^13^. Some strains also produce *C. perfringens* enterotoxin (CPE), a ~35 kDa protein responsible for diarrhea and most of the human food poisoning cases caused by this bacterium^14,15^. The *cpe* gene is located on either the chromosome or large conjugative plasmids and is more prevalent in *C. perfringens* toxinotype F, which is associated with human food poisoning and AAD^13,14^. CPE-positive strains of toxinotypes C and D are uncommon and, to date, there have been no reliable reports of CPE production by toxinotype B strains^16^. Additionally, all *C. perfringens* toxinotypes may also produce an accessory toxin known as β2-toxin encoded by the *cpb2* gene^11,17^. β2-toxin is a pore-forming cytolysin which its role in *C. perfringens* associated enteric diseases still remains to be unraveled^18–20^. The expression of virulence-associated proteins, especially the production of various toxins in *C. perfringens* strains are tightly regulated by specific gene regulatory systems, including VirR/VirS two-component signal transduction system and accessory gene regulator (*agr*) system^21,22^. The *agr* system was first described in *S. aureus* and is comprised of four co-transcribed genes; *agrB*, *agrD*, *agrC*, and *agrA*^23,24^. Via a complex quorum-sensing mechanism, the *agr* system responds to changes in population density and synchronizes the expression of genes involved in virulence, sporulation, and toxin production^25^. The genome of *C. perfringens* encodes two orthologs of the *S. aureus agr* locus; *agrB* and *agrD* genes. The *agrD* gene encodes the precursor AgrD autoinducing peptide (AIP) which is processed to its active form by the integral membrane endoprotease AgrB. AgrD mediates the quorum sensing signal transduction cascade thus plays a critical role in regulating the production of several toxins, including β-toxin^26–29^.

Herein, we provide the first prevalence data of *C. perfringens* in Iranian hospitalized patients suffering from AAD. The molecular epidemiology of *C. perfringens* isolated from this population was characterised by toxinotyping and by PCR amplification of major toxin-encoding genes (*cpa*, *cpb*, *cpb2*, *etx*, *iap*, *cpe*, *netB*) and toxin regulatory genes (*virR*, *virS*, *agrB* and *agrD*). Finally, due to the critical role of AgrD in the regulation of toxin gene expression in *C. perfringens*, a PCR-based sequencing approach was used to investigate amino acid sequence diversity across all typed isolates.

## Materials and Methods

### Study population and fecal sample collection

A total of 2280 consecutive fecal specimens were collected from 2319 patients submitted for routine *C. difficile* testing, who were referred from 54 different hospitals and medical centers to the Research Institute for Gastroenterology and Liver Diseases (RIGLD) in Tehran, Iran, from August 2011 to October 2017. The freshly collected stool specimens were immediately delivered to the Anaerobic Laboratory for testing the presence of *C. perfringens* within 2 hours of collection. Indications for AAD were based on either clinical suspicion or daily production of ≥3 loose or watery stools for at least 2 consecutive days^3^. In addition, AAD was defined as onset of diarrhea after antibiotic therapy given concurrently or discontinued at a maximum of 4 weeks preceding diarrhea. A standardized questionnaire including demographic data, medication history, and underlying health condition was recorded for all patients. The study protocol was approved by the Ethical Review Committee of RIGLD at Shahid Beheshti University of Medical Sciences (Project No. IR.SBMU.RIGLD.REC.1396.185). All experiments were performed in accordance with relevant guidelines and regulations recommended by the institution and informed consents were obtained from all subjects and/or their legal guardians prior to sample collection.

### Culture and isolation of *C. perfringens*

For each sample of feces, approximately 0.5 to 1 g was homogenized in 9 ml of sterile phosphate-buffered saline (PBS) and heat treated in a 70 °C water bath for 20 minutes^30^. Subsequently, 100 μl of the suspension was inoculated onto egg yolk agar (Himedia, India) supplemented with 10% sheep blood and neomycin (0.008 mg/200 ml, Sigma, USA). Culture plates were incubated at 37 °C for 48 h under an anaerobic atmosphere (85% N_2_; 10% CO_2_ and 5% H_2_) generated by Anoxomat^®^ Gas Exchange System (Mart Microbiology BV, Holland). A single bacterial colony from egg yolk agar was sub-cultured on tryptose sulphite cycloserine (TSC) agar supplemented with egg yolk and potassium tellurite and incubated at 37 °C for 48 h under the anaerobic conditions described above. Unusual colonies of *C. perfringens* were identified by growth morphology, Gram’s staining, motility test and biochemicals such as indole production, catalase, oxidase and stormy fermentation of milk. Molecular identification was also performed by PCR amplification of the 16S rRNA gene as previously described^31^.

### Genomic and plasmid DNA preparation

Crude genomic DNA was extracted from the grown colonies on egg yolk agar plates using a boiling method as previously described^32^. Plasmid DNA was purified from the pure colonies after 48 hours of bacterial culture by using GeneJET Plasmid Miniprep Kit (Thermo Fisher Scientific, Lithuania) according to the manufacturer’s instructions. The quality of DNA samples was analyzed by electrophoresis on 0.8% (w/v) agarose gels, then stored at −20 °C until used for PCR experiments.

### Toxinotyping of *C. perfringens* isolates

The presence of toxin genes *cpa*, *cpb*, *cpb2*, *etx*, *iap*, *cpe* and *netB* was examined by PCR using specific primer pairs and based on the conditions presented in Table 1 ^13,33^. PCR was carried out in a final volume of 25 μl reaction mixture composed of 10 µl of Taq DNA Polymerase Master Mix (Ampliqon, Denmark), 0.5 µl of each primer (20 pmol), and 2 µl of template DNA (approximately 200 ng) using a thermocycler (Eppendorf, Hamburg, Germany). Subsequently, an aliquot of each PCR product was analyzed by electrophoresis in a 1.2% agarose gels, followed by ethidium bromide staining and visualization under a UV transilluminator, and then photographed using the gel imaging system (BioDoc-It System, UVP, USA). *C. perfringens* strain RIGLD-33 (accession number: MH997495) and a no-template reaction were used as positive and negative controls in all experiments, respectively.

**Table 1:** Oligonucleotide sequences and PCR conditions of target genes.

Target gene	Primers	Oligonucleotide sequences (5′-3′)	PCR conditions	Product length (bp)	Reference
*cpa* (α-toxin)	CPAlphaF	GCTAATGTTACTGCCGTTGA	94 °C 5 min, 30 cycles(94 °C 1 min; 53 °C 1 min;72 °C 30 sec), 72 °C 5 min	324	^35^
	CPAlphaR	CCTCTGATACATCGTGTAAG			
*cpb* (β-toxin)	CPBetaF3	GCGAATATGCTGAATCATCTA	94 °C 5 min, 30 cycles(94 °C 1 min; 55 °C 1 min;72 °C 30 sec), 72 °C 5 min	195	^35^
	CPBetaR3	GCAGGAACATTAGTATATCTTC			
*cpb2* (β2-toxin)	CPBeta2totalF2	AAATATGATCCTAACCAA**M**AA	94 °C 5 min, 30 cycles(94 °C 1 min; 53 °C 1 min;72 °C 30 sec), 72 °C 5 min	548	^35^
	CPBeta2totalR	CCAAATACT**Y**TAAT**Y**GATGC			
*etx* (ɛ-toxin)	CPEpsilonF	TGGGAACTTCGATACAAGA	94 °C 5 min, 30 cycles(94 °C 1 min; 53 °C 1 min;72 °C 30 sec), 72 °C 5 min	376	^35^
	CPEpsilonR2	AACTGCACTATAATTTCCTTTTCC			
*iap* (ɩ-toxin)	CPIotaF2	AATGGTCCTTTAAATAATCC	94 °C 5 min, 30 cycles(94 °C 1 min; 53 °C 1 min;72 °C 30 sec), 72 °C 5 min	272	^35^
	CpIotaR	TTAGCAAATGCACTCATATT			
*cpe* (enterotoxin)	CPEnteroF	TTCAGTTGGATTTACTTCTG	94 °C 5 min, 30 cycles(94 °C 1 min; 53 °C 1 min;72 °C 30 sec), 72 °C 5 min	485	^35^
	CPEnteroR	TGTCCAGTAGCTGTAATTGT			
*netB* (pore-forming)	JRP6656	CTTCTAGTGATACCGCTTCAC	94 °C 5 min, 30 cycles(94 °C 1 min; 50 °C 1 min;72 °C 30 sec), 72 °C 5 min	738	^13^
	JRP6655	CGTTATATTCACTTGTTGACGAAAG			
*virR*	virR-F	ATAACTGCTTTATGGGATTATATTC	94 °C 5 min, 30 cycles (94 °C 1 min; 47 °C 1 min; 72 °C 30 sec), 72 °C 5 min	277	This study
	virR-R	CTAGTTTATTCATACTCATCTTTAC			
*virS*	virS-F	AAATATCTATAGGATGGAAACTATG	94 °C 5 min, 30 cycles(94 °C 1 min; 47 °C 1 min;72 °C 30 sec), 72 °C 5 min	428	This study
	virS-R	TTCCTTCAATACAGGCTATGTG			
*agrB*	agrBFwd	GATTGAGAATATATCGAAGTTAAT	94 °C 5 min, 30 cycles(94 °C 1 min; 47 °C 1 min;72 °C 30 sec), 72 °C 5 min	589	^29^
	agrBRev	GTAGGTTAGAGTCATACATTGC			
*agrD*	agrD-CpF	CATTTGACTTACCTTGATTAC	94 °C 5 min, 30 cycles(94 °C 1 min; 52 °C 1 min;72 °C 30 sec), 72 °C 5 min	292	This study
	agrD-CpR	TTTGAGCACTTGTTGGTTGG			

M = A or C, Y = C or T.

### Primer design and detection of toxin regulatory genes

The NCBI GenBank database (http://www.ncbi.nlm.nih.gov/genbank) was searched for all available and complete *C. perfringens virR*, *virS* and *agrD* nucleotide sequences. Based on pairwise and multiple nucleotide sequence alignments for each target gene from several *C. perfringens* strains, specific primer pairs were designed from the conserved regions of corresponding genes using CLC Sequence Viewer 8 (https://www.qiagenbioinformatics.com). The nucleotide sequences of *virR*, *virS* and *agrD* genes of *C. perfringens* strain JP838 (CP010994.1, toxinotype A) were used for primer designation. The selected primer target sites were aligned to all available and complete sequences of *C. perfringens* strains using BLAST (http://blast.ncbi.nlm.nih.gov/Blast.cgi). The presence of *virR*, *virS* and *agrD* was examined by PCR as previously mentioned using the designed primers presented in Table 1. *agrB* was also detected using the previously published primers^27^.

### Sequence analysis of *agrD*

For DNA sequencing, PCR amplification was performed in a reaction mixture of 25 µl using primers agrD-CpF and agrD-CpR, yielding amplicons of approximately 292 bp in length. PCR products were purified using the Silica Bead DNA Gel Extraction Kit (Thermo Scientific, Fermentas, USA) followed by sequencing of both forward and reverse strands using an automated sequencer ABI 3730XL (Macrogen, Seoul, Korea). DNA sequences were edited using Chromas Lite v2.5.1 (Technelysium Pty Ltd, Australia) and BioEdit v7.2.5^34^. All the complete *agrD* nucleotide and amino acid sequences were aligned to the *agrD* sequence of *C. perfringens* reference strain FORC_025 (CP013101.1, toxinotype A) as a scaffold sequence after in-frame translation. Single nucleotide variations and amino acid polymorphism of the translated sequences were examined using BioEdit v7.2.5.

### Nucleotide sequence accession numbers

The complete nucleotide sequences of *agrD* genes from the 64 *C. perfringens* isolates characterized in this work were deposited in the NCBI GenBank database under accession numbers MH377337 to MH377400.

### Statistical analysis

Statistical analyses were performed by IBM SPSS Statistics version 21 (Armonk, NY: IBM Corp.). Clinical and molecular data were analyzed by the Chi-square and Fisher’s exact test. A *P* value less than 0.05 was regarded as statistically significant.

## Results

### Prevalence of *C. perfringens* in fecal samples

Overall, AAD was diagnosed in 13.3% (303/2280) of patients examined in this study. A total of 106 isolates of *C. perfringens* were cultured from 2208 fecal specimens (4.8%), with 56 (52.8%) female and 50 (47.2%) male, aged from <1 to 87 years with a mean age of 36.3 years. Of these *C. perfringens-*positive patients, 68 presented with AAD (AAD group) and 38 did not have AAD (non-AAD group). Demographic data on age, gender, duration of diarrhea and underlying diseases for the study groups are summarized in Table 2. A significant association was found between patients aged >50 years and AAD (*P* = 0.001). More than half of the AAD patients and about 60% of patients in the non-AAD group suffered from inflammatory bowel disease (IBD), all of which had ulcerative colitis (UC), except for one patient having Crohn’s disease (CD) in AAD group. Approximately, one-third of AAD patients were receiving metronidazole treatment alone, while 10 (14.6%), 6 (8.8%), 1 (1.5%) and 1 (1.5%) were receiving metronidazole treatment plus either vancomycin, ciprofloxacin, imipenem or ceftriaxone, respectively. Moreover, 5 (7.3%) AAD patients were on the combination of vancomycin plus either ciprofloxacin, imipenem, meropenem or amphotericin B. The frequency of various patterns of the antibiotics used in *C. perfringens*-positive patients with AAD is presented in Table 3.

**Table 2:** Demographic and clinical characteristics of 106 *C. perfringens*-positive patients.

Patient characteristics	AAD group (n=68)	Non-AAD group (n=38)	Total (%) (n=106)	*P* value^*^
**Age (years)**				
1–10	5 (7.4)	8 (21)	13 (12.3)	**0.001**
11–20	2 (2.9)	2 (5.2)	4 (3.8)	
21-30	18 (26.5)	12 (31.7)	30 (28.3)	
31-40	9 (13.2)	9 (23.7)	18 (16.9)	
41-50	9 (13.2)	4 (10.5)	13 (12.2)	
>50	25 (36.8)	3 (7.9)	28 (26.5)	
**Gender**				
Female	36 (53)	20 (52.6)	56 (52.8)	1
Male	32 (47)	18 (47.4)	50 (47.2)	
**Duration of diarrhea**				
2-3 days	51 (75)	25 (65.8)	76 (71.7)	0.37
>3 days	17 (25)	13 (32.2)	30 (28.3)	
**Defecation (times/day)**				
3–5	45 (66.2)	20 (53)	65 (61.4)	0.21
5–8	13 (19.1)	14 (36.5)	27 (25.4)	
8–10	9 (13.2)	4 (10.5)	13 (12.3)	
>10	1 (1.5)	0	1 (0.9)	
**Underlying diseases**				
Cancer	13 (19.1)	4 (10.5)	17 (16)	0.59
Renal failure	2 (2.9)	0	2 (1.9)	
Dementia	2 (2.9)	0	2 (1.9)	
Immunodeficiency disorders	2 (2.9)	1 (2.7)	3 (2.8)	
Inflammatory bowel disease (IBD)	37 (54.5)	23 (60.5)	60 (56.7)	
Inflammatory bowel syndrome (IBS)	1 (1.5)	1 (2.6)	2 (1.9)	
**Hospital wards**				
Internal	9 (13.2)	3 (7.9)	12 (11.3)	0.21
Gastroenterology	31 (45.6)	16 (42.1)	47 (44.3)	
Infectious	2 (2.9)	2 (5.2)	4 (3.8)	
Oncology	10 (14.7)	1 (2.7)	11 (10.4)	
Intensive care unit (ICU)	1 (1.5)	1 (2.7)	2 (1.9)	
Coronary care unit (CCU)	1 (1.5)	0	1 (0.9)	
Bone marrow transplantation (BMT)	2 (2.9)	1 (2.7)	3 (2.8)	
Nephrology	1 (1.5)	1 (2.7)	2 (1.9)	
Orthopedic	1 (1.5)	0	1 (0.9)	
Out-patient	10 (14.7)	13 (34.2)	23 (21.8)	

AAD, antibiotic-associated diarrhea.

*Statistical analysis was performed by Chi-square and Fisher’s exact tests. The *P* value in bold indicates a significant association between AAD and patients’ age over 50 years.

**Table 3:** The frequency of various patterns of antibiotic use in *C. perfringens*-positive patients with AAD.

Patterns of antibiotic use	AAD group (n = 68)
Ceftriaxone	3 (4.4)
Ciprofloxacin	3 (4.4)
Metronidazole	23 (33.6)
Meropenem	2 (2.9)
Chloramphenicol	1 (1.5)
Imipenem	2 (2.9)
Tinidazole	1 (1.5)
Metronidazole + Vancomycin	10 (14.6)
Metronidazole + Ciprofloxacin	6 (8.8)
Metronidazole + Imipenem	1 (1.5)
Metronidazole + Ceftriaxone	1 (1.5)
Ciprofloxacin + Amikacin	1 (1.5)
Ciprofloxacin + Ceftazidime	1 (1.5)
Ciprofloxacin + Ceftriaxone	1 (1.5)
Ciprofloxacin + Chloramphenicol	1 (1.5)
Ciprofloxacin + Ceftriaxone	1 (1.5)
Ciprofloxacin + Trimethoprim-Sulfamethoxazole	1 (1.5)
Ciprofloxacin + Ceftazidime + Azithromycin	1 (1.5)
Ciprofloxacin + Meropenem + Trimethoprim-Sulfamethoxazole	1 (1.5)
Vancomycin + Ciprofloxacin	2 (2.9)
Vancomycin + Imipenem	1 (1.5)
Vancomycin + Meropenem	1 (1.5)
Vancomycin + Amphotericin B	1 (1.5)
Azithromycin + Cefixime	1 (1.5)
Azithromycin + Cefixime + Metronidazole	1 (1.5)

AAD, antibiotic-associated diarrhea.

### Prevalence of toxin genes of *C. perfringens* isolates

The prevalence of toxin-encoding genes and toxinotypes of *C. perfringens* isolates among AAD and non-AAD groups are shown in Table 4. In all 106 *C. perfringens* isolates, PCR revealed the presence of the *cpa* gene encoding the species-specific phospholipase C or α-toxin. None of the *C. perfringens* isolates harbored *cpb*, *etx* and *iap* genes which encode β-, ɛ-, and ɩ-toxins, respectively. The presence of *cpb2* gene was identified in 14 (20.6%) isolates in the AAD group and in 10 (26.3%) isolates in the non-AAD group. The *cpe* gene was detected in 9 (13.2%) isolates in the AAD group and in 8 (21%) isolates in the non-AAD group. Additionally, none of the *C. perfringens* isolates carried the *netB* gene. No significant association was observed between the presence of any of these toxins and AAD (*P* > 0.05).

**Table 4:** The prevalence of toxin encoding genes, toxin regulatory genes and toxinotypes in *C. perfringens*-positive patients with AAD and without AAD.

Toxin gene	AAD group (n = 68)	Non-AAD group (n = 38)	Total (%) (n = 106)	*P* value^*^
*cpa*	68 (100)	38 (100)	106 (100)	
*cpb*	0	0	0	
*cpb2*	14 (20.6)	10 (26.3)	24 (22.6)	0.66
*etx*	0	0	0	
*iap*	0	0	0	
*cpe*	9 (13.2)	8 (21)	17 (16)	0.44
*netB*	0	0	0	
**Toxinotype**				
A	59 (86.8)	30 (78.9)	89 (84)	0.43
B	0	0	0	
C	0	0	0	
D	0	0	0	
E	0	0	0	
F	9 (13.2)	8 (21)	17 (16)	0.44
G	0	0	0	

AAD, antibiotic-associated diarrhea.

*Statistical analysis was performed by Chi-square and Fisher’s exact tests.

### Association of *C. perfringens* toxinotypes with AAD

*C. perfringens* type A (*cpa*-positive) was the most prevalent toxinotype, accounting for 84% (89/106) of isolates. New *C. perfringens* toxinotypes F (α-toxin positive, *cpe*-positive) was found in 16.0% (17/106) of all isolates. Interestingly, this toxinotype was almost equally prevalent in both AAD and non-AAD groups as shown in Table 4. *C. perfringens* types B, C, D, E and G were not identified. Additionally, 28.8% (17/59) of the *C. perfringens* type A strains were found to be *cpb2*-positive, while 41.2% (7/17) of the type F strains were seen to be *cpb2*-positive mostly in AAD group. There was no significant association between different toxinotypes of *C. perfringens* strains and AAD (*P* > 0.05).

### Distribution of toxin regulatory genes in *C. perfringens* toxinotypes

Toxin regulatory genes *virR* and *virS* were simultaneously detected in 60 (56.6%) isolates of *C. perfringens*. Similarly, 71 (67%) isolates were found to harbor *agrB* and *agrD* genes. The *virR/virS* regulon was present in 52 (88.1%) type A isolates and 8 (47%) type F isolates. In addition, simultaneous presence of *agrB* and *agrD* was seen in 57 (96.6%) and 14 (82.3%) isolates with toxinotypes A and F, respectively.

### AgrD sequence diversity of *C. perfringens* isolates

*agrD* PCR products from 64 *C. perfringens* strains isolated from patients in AAD (51 isolates) and non-AAD (13 isolates) groups were subjected to nucleotide sequencing. The obtained sequences were manually edited and trimmed, and 7 *agrD* sequences that did not meet the required quality to be incorporated into polymorphism analysis were removed from the study. Comparative analysis with sequences available in the NCBI GenBank database revealed all *C. perfringens* sequences from this study shared >97% sequence homology with the *agrD* gene of the *C. perfringens* strain FORC_025 (CP013101.1, toxinotype A).

Comparative analysis with the AgrD amino acid sequence of FORC_025 found all study AgrD peptides to be highly conserved, sharing 97 to 100% sequence identity (Supplementary Fig. 1). Notably, there was only one amino acid change at position 24 from alanine (A) to valine (V) in the N-terminal amphipathic region of AgrD peptide among all analyzed *C. perfringens* isolates. The putative amino acids predicted to form the thiolactone/lactone ring structure of the mature AIP were also conserved as CLWFT in all isolates, except for four isolates, three in AAD group and one in the non-AAD group, which presented CIWFT as the ring structure. A highly conserved proline (P) residue in the C-terminal charged region was found in all AgrD peptides of *C. perfringens* isolates. No significant difference was observed between AgrD peptide sequences of *C. perfringens* in the AAD and non-AAD groups (Supplementary Fig. 1).

## Discussion

AAD is a complex and debilitating disorder, which results from dysbiosis of the protective gut microbiota. Disruption to the colonic microbiota is primarily due to exposure to antibiotic therapy, but other known risk factors for AAD include the use of proton pump inhibitors, exposure to pathogens in high-risk environments, medical procedures, senescence, comorbidity, IBD and immunosuppression^35^. *C. perfringens* forms part of the normal gut flora of healthy humans in small numbers up to 10^3^ colony forming units (cfu) per gram. Ingestion of large numbers (>10^8^ organisms) of enterotoxigenic *C. perfringens* followed by their multiplication and release of CPE enterotoxin in the small intestine can develop AAD if allowed to overgrow^36^. Each toxinotype of *C. perfringens* is associated with histotoxic and enterotoxic diseases in humans and animals, and in particular, *C. perfringens* CPE-positive toxinotype A (currently called *C. perfringens* type F strains) are considered an important causative agent in cases of AAD^13,35,37^.

This study investigated the prevalence and molecular epidemiology of *C. perfringens* isolated from Iranian hospitalized patients suffering from AAD. Overall, 13.3% of the test population presented with AAD, and a total of 106 (4.8%) *C. perfringens* strains were isolated from 2208 fecal samples collected over a 6 year period, in which 68 isolates were obtained from AAD patients and 38 from non-AAD. We believe this is the first report for the prevalence of *C. perfringens* among Iranian patients with diarrheal illnesses. Previous studies from Germany^38^ and India^8^ reported *C. perfringens* prevalence in stool of 4.1% and 8.6%, respectively. Recently, Young *et al*. detected the *C. perfringens* toxin in 14 of 135 (10.4%) fecal specimens of patients suspected of having AAD^2^. Interestingly, the prevalence of *C. perfringens* among AAD patients in our study was very high and found to be about 22.4%. This finding highlights the significance of a screening test for detection of this pathogen as an important alternative to *C. difficile* in the development of AAD.

We observed *C. perfringens*-infected patients that aged over 50 years were at higher risk to present AAD (*P* = 0.001) than in younger patients. In a previous study by Forward *et al*. higher levels of CPE toxin were found in patients aged over 60 years^39^. They suggested that testing for CPE would be of value in cases of diarrhea in patients aged over 60 years, regardless if the patient has a history of antibiotic treatment. In our study, more than half of the AAD patients positive for *C. perfringens* suffered from IBD, although this was not statistically significant. However, it has been suggested that clostridia other than *C. difficile*, especially enterotoxigenic *C. perfringens*, may play a significant role in the clinical course of IBD^40^. Notably, 61.5% of the AAD cases had received metronidazole alone or in combination with other antibacterial therapies, more frequently vancomycin. These data are in agreement with another study by Joshy *et al*. from India, where 48.6% of AAD patients had received metronidazole and/or vancomycin treatment^8^. In both AAD and non-AAD groups, nearly half of the patients positive for *C. perfringens* were located in gastroenterology wards. However, about 91% of the patients positive for *C. perfringens* from oncology wards had AAD. This finding is in agreement with the data from the study by Joshy *et al*., in which 100% of the enterotoxigenic *C. perfringens* isolates from AAD cases came from oncology medical wards^8^.

In this study, CPE*-*positive *C. perfringens* was detected in 16% of all patients. Notably, 13.2% of the patients in the AAD group were *cpe*-positive, although not statistically different when compared with the non-AAD group. Previous studies found CPE in 0.14%^41^, 3.3%^3^, 6.4%^38^ and 8%^30^ of cases with AAD by using either an enzyme-linked immunosorbent assay (ELISA) or more sensitive molecular methods. In comparison, other studies reported that the prevalence of CPE-positive *C. perfringens* was higher, ranging from 2.5 to 31%, in the cases of sporadic diarrhea (SD) than AAD^39,42,43^. In the above studies, CPE was detected using the ELISA, reversed passive latex agglutination (RPLA) and/or enzyme immunoassay (EIA) tests, thus the differences in CPE prevalence may be due to testing methodology or more likely, differing strain/toxinotype epidemiology in different geographical regions.

As anticipated, all isolates contained the *cpa* gene with toxinotype A accounting for 84% of isolates. Toxinotype A was detected with higher prevalence in AAD group (86.8%) than non-AAD group (78.9%). It has been reported that 1 to 5% of *C. perfringens* type A strains also produce CPE^41^. Previously, these CPE-positive strains of *C. perfringens* were referred to as type A. However, recently Rood *et al*. developed a new toxin-based typing scheme whereby *C. perfringens* is now classified into 7 toxinotypes, A to G^13^. Based on this new scheme, all CPE-positive strains of *C. perfringens* type A are now renamed as *C. perfringens* type F strains. In our study, *C. perfringens* type F strains were found in 16% of the isolates, and 13.2% of the patients in the AAD group carried this toxinotype. This provides some evidence for the importance of CPE-producing type F strains in the development of AAD.

As expected, *C. perfringens* type B, C, D and E strains were not detected in this population. Moreover, certain strains of *C. perfringens* that produce a plasmid-encoded pore-forming toxin called NetB have been reported to cause necrotic enteritis in poultry^44^. The infected chickens may present various clinical symptoms such as diarrhea, ruffled feathers, anorexia, depression and occasionally sudden death without any warning clinical signs^45^. As mentioned earlier, these *netB*-positive isolates have recently been described as toxinotype G strains. As expected, none of our human isolates of *C. perfringens* carried the *netB* gene.

Beta2-toxin (CPB2) is another plasmid-borne toxin of *C. perfringens* encoded by *cpb2* gene, which has 15% amino acid sequence identity with CPB^46^. *cpb2*-positive *C. perfringens* strains have been identified from a variety of animals suffering from enteric diseases, including domestic and wild animals. Moreover, Fisher *et al*. detected the *cpb2* gene in <15% of food poisoning isolates by PCR^47^. They also reported that >75% of AAD/SD isolates, which usually carry a plasmid-borne *cpe* gene, were also found to be positive for *cpb2* gene and suggested that CPB2 may be involved in CPE-associated AAD/SD as an accessory toxin of *C. perfringens*. However, further experimental evidence is needed to support the role of CPB2 toxin in the pathogenesis of *C. perfringens* induced AAD. Notably, in our study, 41.2% of the *C. perfringens* type F strains that carry the *cpe* gene were also found to be *cpb2*-positive, and more common in the AAD group.

The two-component system (TCS) of pathogenic clostridia, which comprises the VirS membrane sensor histidine kinase and its cognate VirR response regulator, regulates the production of several toxins including CPB, as well as many extracellular enzymes^21,48^. The mutation or inactivation of either the *virS* or *virR* gene results in an altered toxin production compared to the wild-type strain of *C. perfringens*^48,49^. Although the VirS/VirR regulon is located on the chromosome, it can regulate genes carried on both the chromosome and the plasmid^21^. In addition, many bacteria regulate gene expression in response to their population or cell density in a QS based manner. The Agr-like QS system in *C. perfringens* functions as a cell-to-cell communication system and has been shown to be important for the regulation of virulence genes in this species^21,29^. Initial studies have shown that the QS system can regulate the production of a number of extracellular toxins *in vitro*, including CPA, PFO, CPB, ETX, CPB2, CPE, and NetB^22,25,47,50^. In *C. perfringens*, the AgrD is first processed to the active AIP by the AgrB membrane endoprotease and then released into the extracellular milieu. However, data obtained from a genome analysis showed that *C. perfringens* lacks the AgrA/AgrC as the TCS upstream or downstream of *agrBD* that responds to the AIP in *S. aureus*^25^. There is also some evidence, but not firmly proven, indicating that the VirS/VirR regulatory system may corresponds to AgrA/AgrC in *C. perfringens*^21^. In our study, we identified the *virR/virS* regulon in 56.6% of the isolates, while the *agrB* and *agrD* genes were detected in 67% of the *C. perfringens* isolates simultaneously. Moreover, because of the critical function of AgrD in the modification of gene expression in *C. perfringens*, we investigated its amino acid sequence variations among the studied isolates. This is the first study, however, to report the prevalence of *agrD* and its genetic diversity in *C. perfringens* strains. Our results showed that 67% of *C. perfringens* strains harbored *agrD*. Notably, the majority of *agrD*-positive strains (75%) were found in the AAD group. Moreover, the data obtained from the sequence analysis demonstrated very high conservation of AgrD in Iranian *C. perfringens* strains, irrespective of toxinotype, particularly in the thiolactone/lactone ring structure containing CLWFT motif. However, a few strains contained CIWFT in their mature AIP ring structure, mostly in the AAD group. Recently, bioinformatic analysis of the genome sequences of pathogenic clostridia and different *agr* types of *S. aureus* has revealed significant variations in the amino acid residues in the thiolactone/lactone ring structure of the putative AgrD peptides^46^. Further studies are needed to ascertain the functional consequences of these underlying variations in AgrD.

In summary, the high prevalence of *C. perfringens* in AAD patients suggests that it is a potentially important cause of infective AAD in the Iranian population, especially if the stool is negative for other pathogens such as *C. difficile*. In addition, enterotoxigenic toxinotype F isolates that produce CPE were detected in a large proportion of the study population. We acknowledge that this study focused solely on the prevalence of *C. perfringens* and its toxinotypes, as such we could not assess other bacterial agents that can cause AAD, such as *C. difficile*, *S. aureus* and *K. oxytoca*. Moreover, we also did not rule out the possibility of foodborne diarrhea caused by *C. perfringens*. However, our findings suggest that routine diagnostic laboratories in Iran should consider screening for *C. perfringens* in cases of suspected AAD, particularly in patients over 50 years. Our results also showed that the majority of patients with AAD carried *agrD*-positive *C. perfringens*, however, further studies are required to evaluate its clinical significance in the pathogenesis of AAD caused by *C. perfringens*. Finally, extended monitoring of the true burden of *C. perfringens* AAD in both hospital patients and in the community by surveillance and epidemiological studies could markedly improve the efficacy of infection prevention and control measures.

## Supplementary information

Supplementary Figure 1
