# Supplementary material for: Prevalence and characterization of Clostridium perfringens toxinotypes among patients with antibiotic-associated diarrhea in Iran
Source: Sci Rep. 2019 May 24;9:7792. doi: 10.1038/s41598-019-44281-5 (PMC6534674; doi:10.1038/s41598-019-44281-5)
Supplement: Supplementary file 1 — Supplementary Figure 1 [file 41598_2019_44281_MOESM1_ESM.pdf]

Prevalence and characterization of *Clostridium perfringens* toxinotypes among patients with antibiotic-associated diarrhea in Iran

Masoumeh Azimirad, Fatemeh Gholami, Abbas Yadegar, Daniel R. Knight, Sharareh Shamloei, Hamid Asadzadeh Aghdaei & Mohammad Reza Zali

Supplementary information

Supplementary Figure 1:

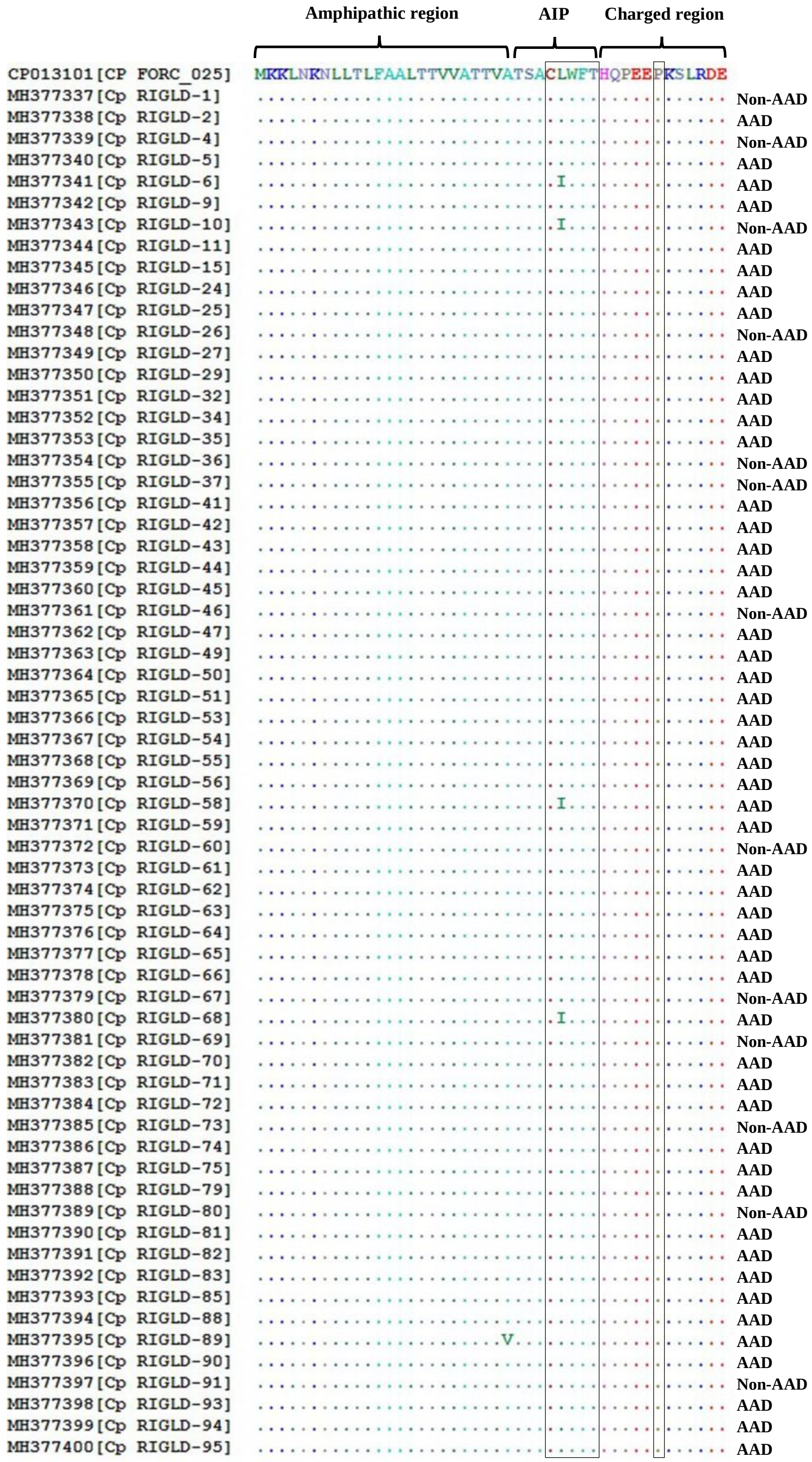

Supplementary Figure 1. Complete amino acid sequence alignment of AgrD from 64 *C. perfringens* isolates from patients in antibiotic-associated diarrhea (AAD) group and non-AAD group. The AgrD amino acid sequence of *C. perfringens* strain FORC_025 (CP013101.1) as the reference strain is shown on the top line. The peptides are split into three regions representing the N-terminal amphipathic region, the autoinducing peptide (AIP) encoding region and the C-terminal charged region. The putative amino acid motif to form the thiolactone/lactone ring structure of the mature AIP and a highly conserved proline residue found in all AgrD peptides are surrounded in boxes.
